# Genetic Association of Olanzapine Treatment Response in Han Chinese Schizophrenia Patients

**DOI:** 10.3389/fphar.2019.00177

**Published:** 2019-03-04

**Authors:** Wei Zhou, Yong Xu, Qinyu Lv, Yong-hui Sheng, Luan Chen, Mo Li, Lu Shen, Cong Huai, Zhenghui Yi, Donghong Cui, Shengying Qin

**Affiliations:** ^1^Key Laboratory for the Genetics of Developmental and Neuropsychiatric Disorders, Bio-X Institutes, Ministry of Education, Shanghai Jiao Tong University, Shanghai, China; ^2^Department of Psychiatry, First Hospital of Shanxi Medical University, Taiyuan, China; ^3^Shanghai Mental Health Center, Shanghai Jiao Tong University School of Medicine, Shanghai, China; ^4^Shandong Mental Health Center, Jinan, China; ^5^Shanghai Key Laboratory of Psychotic Disorders, Shanghai, China; ^6^The Third Affiliated Hospital, Guangzhou Medical University, Guangzhou, China

**Keywords:** olanzapine, polymorphism, schizophrenia, pharmacogenetics, biomarker, association study

## Abstract

Olanzapine, a second-generation antipsychotic medication, plays a critical role in current treatment of schizophrenia (SCZ). It has been observed that the olanzapine responses in schizophrenia treatment are different across individuals. However, prediction of this individual-specific olanzapine response requires in-depth knowledge of biomarkers of drug response. Here, we performed an integrative investigation on 238 Han Chinese SCZ patients to identify predictive biomarkers that were associated with the efficacy of olanzapine treatment. This study applied HaloPlex technology to sequence 143 genes from 79 Han Chinese SCZ patients. Our result suggested that there were 12 single nucleotide polymorphisms (SNPs) had significant association with olanzapine response in Han Chinese SCZ patients. Using MassARRAY platform, we tested that if these 12 SNPs were also statistically significant in 159 other SCZ patients (independent cohort) and the combined 238 SCZ patients (composed of two tested cohorts). The result of this analysis showed that 2 SNPs were significantly associated with the olanzapine response in both independent cohorts (rs324026, *P* = 0.023; rs12610827, *P* = 0.043) and combined SCZ patient population (rs324026, adjust *P* = 0.014; rs12610827, adjust *P* = 0.012). Our study provides systematic analyses of genetic variants associated with olanzapine responses of Han Chinese SCZ patients. The discovery of these novel biomarkers of olanzapine-response will facilitate to advance future olanzapine treatment specific for Han Chinese SCZ patients.

## Introduction

Schizophrenia (SCZ) is a severe chronic neuropsychiatric illness. According to a survey carried out in 33 countries, about 15 out of 100,000 individuals were suffering from SCZ globally (McGrath et al., 2004). These SCZ patients were estimated to have higher risk of death (about 2.5 times more) compared to healthy individuals (McGrath et al., 2004; Saha et al., 2007). Patients with SCZ disorder require long-term treatments to prevent themselves from illness progression or symptom relapse (Howes et al., 2015; Chong et al., 2016). Although the development of SCZ has long been regarded as caused by a combination of genetic and environmental factors, detailed pathophysiological mechanism of SCZ still remains unclear.

Second-generation antipsychotic (SGA) medications are widely considered as the most advanced and effective treatment for SCZ patients nowadays. These SGA includes olanzapine (OLA), risperidone and quetiapine (Owen et al., 2016). However, SCZ patients who received SGA treatments often experienced severe adverse drug reactions (ADRs) (Zhang and Malhotra, 2011). In fact, current SGA therapies could be seen as a subjective “trial and error” process. For some of the SCZ patients, these SGA treatments did not exert any therapeutic effects on their symptoms. This individual-specific response to SGA may be caused by the association between individual-specific genetic variation and the efficacy of SGAs (Zhang and Malhotra, 2011). In order to address such specificity of SGA response across different patients, scientists in pharmacogenomics field is now exploring the possibility of predicting drug response using individual-specific genetic signatures (Arranz and de Leon, 2007).

Olanzapine is one of the most commonly used SGAs. It has been shown to have relatively superior efficacy in various clinical trials compared to other SGAs (Lieberman et al., 2005; Leucht et al., 2009). It has been reported that OLA treatment had relatively low extrapyramidal side effects and better efficacy to minimize negative symptoms of SCZ patients when it is used in clinically effective doses (Meltzer, 1999). OLA binds to serotonin type 2 (5-HT2) and dopamine (D2) receptors with high affinity in patients’ body. Diphosphate glucuronosyltransferases (UGT), a member of cytochrome P450 family and flavin-containing monooxygenase 1 (FMO1), catalyze the oxidative hepatic metabolism process of OLA (Ring et al., 1996; Kassahun et al., 1997; Linnet, 2002). On the other hand, due to the heterogeneity in different SCZ patients, not all patients respond to OLA treatment adequately well as we expected. Some patients who received OLA therapy even experienced severe adverse side effects that resulted in non-compliance with drug treatment (Zhang and Malhotra, 2011; Musil et al., 2015). If these SCZ patients with no other effective therapies specific for them they would have to face the coming disease progression, relapses and potential long-term hospitalizations (Robinson et al., 1999; King et al., 2014).

Although numerous studies have been performed on the factors that influence the therapeutic efficacy of OLA, there were very few of them focused on the individual-specific genetic biomarkers of OLA response (Söderberg and Dahl, 2013). In comparison, earlier attempts to search for biomarkers of OLA response focused mainly on the relationship between OLA response and its metabolic pathways, including glucuronidation, hydroxylation, *N*-demethylation and N-oxidation pathways (Laika et al., 2010; Haslemo et al., 2012; Mao et al., 2012; Söderberg et al., 2013; Brandl et al., 2015). A number of genetic variants, including *UGT2B10* rs61750900 (UGT2B10^∗^2) (Erickson-Ridout et al., 2011), *CYP1A2* rs762551 (CYP1A2^∗^1F) (Laika et al., 2010), DRD3 rs6280 (Adams et al., 2008), *AHR* rs4410790 (Söderberg et al., 2013), *FMO3* K158–G308, *FMO1* rs12720462 (FMO1^∗^6) and *FMO1* rs7877 have been reported to play important roles to influence OLA metabolism (Soderberg et al., 2013). In addition, the drug response and the pharmacokinetics of OLA have also been found to associate with genetic elements that are not directly involved in the metabolic pathway of OLA (Lin et al., 2006; Meary et al., 2008; Cabaleiro et al., 2013; Yu et al., 2018). For example, *P*-glycoprotein, a membrane protein that pumps foreign substances out of cells and is regarded as element that is not directly related to OLA metabolic pathway, affects the penetration of OLA into the central nervous system (Lin et al., 2006). These discoveries suggested that a comprehensive study of OLA response requires clear understanding of the complicated biological network that is composed of enzymes involved in drug metabolism, drug transportation and drugs targeted receptors.

In this study, we investigated the associations between SNPs in 143 genes and the OLA response of 79 Han Chinese SCZ patients using target-sequencing technology. The newly found biomarkers was considered as genetic signature of drug responses to 8-week treatment with OLA and were validated in the other independent Han Chinese SCZ patient cohort.

## Materials and Methods

### Subjects

In this study, we collected 2 independent sets of OLA response data from Han Chinese SCZ patients in order to validate our discoveries. We named the first set as ‘discovery cohort’ and other one as ‘independent cohort.’ The demographics and clinical details of the both sets of patients are demonstrated in Table 1.

**Table 1:** Demographic and clinical details of patients suffering from schizophrenia in three cohorts.

Baseline characteristics^a^	Discovery cohort		*P*-value	Independent cohort		*P*-value	Total cohort		*P*-value
	Good	Poor		Good	Poor		Good	Poor
	responders	responders		responders	responders		responders	responders	
Number	38	41	-	119	40	–	157	81	–
Age (years)	45.4 ± 19.7	39.9 ± 16.2	0.15	39.5 ± 16.77	36.68 ± 15.4	0.35	41.1 ± 17.6	39.1 ± 16.2	0.41
Gender (male/female)	17/21	20/21	0.72	56/63	19/21	0.96	73/84	39/42	0.78
BMI, kg m^-2^	21.6 ± 3.5	21.9 ± 3.6	0.84	22.8 ± 3.8	22.5 ± 3.4	0.71	22.5 ± 3.8	22.1 ± 3.4	0.46
PANSS total score T0	78.4 ± 18.0	73.8 ± 12.2	0.32	82.6 ± 23.3	78.0 ± 21.1	0.28	81.5 ± 22.1	75.9 ± 17.4	0.051
PANSS total score T1	45.5 ± 6.9	58.3 ± 8.4	**<0.001**	44.2 ± 10.1	63.3 ± 14.4	**<0.001**	44.5 ± 9.4	60.9 ± 12.1	**<0.001**


*BMI, body mass index; T0 – baseline measurement, T1 – follow-up measurement (8 weeks). Bold font indicates statistically significant values (P < 0.05).Values are shown as means ± standard deviation.*

*^a^Gender was analyzed by χ^2^ test and other characteristics were analyzed by Student’s t-test*.

The discovery cohort was composed of 79 recruited Han Chinese SCZ patients who had been treated with OLA from the Shanghai Mental Health Center of China. It comprised 37 males and 42 females. The mean age of them was 43.1 ± 18.3 years old (Table 1). SCZ of the patients was diagnosed according to the criteria of the Diagnostic and Statistical Manual of Mental Disorders, Fourth Edition (DSM-IV) and confirmed by at least two experienced psychiatrists. Patients who had physical complications or other substance abuse were excluded from this investigation. Our analysis only considered patients who had not been previously treated with atypical antipsychotics and had not received any medication for more than 4 weeks before their enrollment in this study.

The independent cohort contained 159 recruited Han Chinese SCZ patients who were undergoing OLA monotherapy from the Shanghai Mental Health Center of China and the First Hospital of Shanxi Medical University. They were composed of 75 males and 84 females. The mean age of independent cohort was 38.5 ± 16.4 years (Table 1). This cohort of patients was used to validate the novel biomarkers found by the analysis of discovery cohort. The selection criteria were similar to the discovery cohort. Finally, patient data from discovery cohort and independent cohort were combined in order to gain a greater power in statistical analysis. Therefore, a total of 238 patients were used to perform another validation of the newly found biomarkers.

### Clinical Assessment

Clinical effects of patients were evaluated using the Positive and Negative Syndrome Scale (PANSS) by two fully qualified psychiatrists during the 8 weeks of OLA treatment. The inter-rater reliability between the two raters was found to be high since the intraclass correlation coefficients (ICCs) was larger than 0.8. Based to Obermeier’s method, patients were classified as good responders (reduction of PNASS score ≥ 50%) and poor responders (reduction of PNASS score < 50%) for analysis (Obermeier et al., 2010). The initial daily dose of OLA was 10 mg per day and then it gradually increased to 15 mg per day within the 1st week. After that, the dosage was adjusted based on individual tolerance to the treatment. During the medication period, nursing staff closely monitored any medication compliance occurred in patients. No other drugs were administered when OLA monotherapy was performed, except for sennoside for constipation, flunitrazepam or lorazepam for acute insomnia and biperiden for any extrapyramidal side effects.

### Ethics Statement

The study was approved by the Ethical Committee of Human Genetic Resources in Shanghai, China. All subjects or their legal guardians understood the procedure and had given written informed consent to their participation in this study according to the Declaration of Helsinki (Human, 1999).

### Targeted Genes and Capture Design

One hundred and forty-three genes were selected for targeted sequencing were based on their involvement in drug metabolism (including genes encode for drug-metabolizing enzymes, drug-transporting enzymes and the receptors mediating drug responses) from PharmGKB database^1^ and the relevant literatures (Arranz et al., 2011; Li and Bluth, 2011; Arranz et al., 2016) that reported potential genes related to SGAs efficacy. We aimed to study these genes in order to investigate novel biomarkers of OLA response. The details of these 143 genes are listed in Supplementary Table S1.

Sequencing probes for the 143 targeted genes were designed using Agilent’s SureDesign tool ^2^. Targeted regions of these genes of interest included their coding regions ± 10 bp and untranslated regions (UTR) according to information from RefSeq, Ensembl, CCDS, and GENCODE databases (Harrow et al., 2012; Pruitt et al., 2014; Cunningham et al., 2015).

### Library Preparation and Next Generation Sequencing

Genomic DNA was extracted from whole blood using a QIAamp DNA Blood Mini Kit (Qiagen GmbH, Hilden, Germany). The quantity and quality of the genomic DNA were measured by Nanodrop 2000 (Thermo Scientific, United States). Then we adjusted the genomic DNA to a final concentration of 100 ng/μl with high-purity water and stored at -20°C. Libraries were prepared with a HaloPlex Target Enrichment System Kit (Agilent Technologies, Santa Clara, CA, United States) following the manufacturer’s instructions. Libraries were then quantified using the Agilent 2100 Bioanalyzer (Agilent Technologies). Sequencing was performed with the HiSeq 2500 platform (Illumina, San Diego, CA, United States) using paired-end libraries (2 × 101-bp).

Raw data were processed following standard protocols used in earlier reports (Gaynor et al., 2016). In short, raw image files were first converted to the FASTQ format and the reads were aligned to the human reference genome (hg19, GRCh37). SNPs were identified according to GATK standard hard filtering parameters (DePristo et al., 2011). On average, 99% of reads covered >80× and 81% >200×, which suggested that the coverage was sufficiently high to detect variants with appropriate sensitivity. The program ANNOVAR was used to annotate SNVs that covered >20× according to the information from Ensembl Variation, dbSNP, and 1000genome database (Sherry et al., 2001; Abecasis et al., 2010; Wang et al., 2010; Flicek et al., 2012). Subsequently, individual and SNP-level quality controls were performed using PLINK (v1.07) software (Purcell et al., 2007). Data cleaning was performed according to the following criteria: genotypic call rate < 95%, Hardy–Weinberg equilibrium (HWE) < 0.001, and minor allele frequency (MAF) < 0.01. After we conducted quality controls of the sequences, there were 77 individuals (38 good responders and 39 poor responders) and 807 SNPs remained in our data for later analysis.

### Validation Trial

We identified 12 SNPs that were significantly associated with OLA response using discovery cohort. Similar analysis was performed using both independent cohort and patient samples with two cohort combined in order to validate these newly found SNPs. These 12 SNPs were genotyped using the Sequenom MassARRAY platform (Agena Bioscience, San Diego, CA, United States) following manufacturer’s instructions. MassARRAY primers were designed using a semi-automated software Assay Design Suite v2.0^3^. The primer sequences are listed in Supplementary Table S2. Data cleaning was performed according to the following criteria: genotypic call rate < 95%, Hardy–Weinberg equilibrium (HWE) < 0.001, and minor allele frequency (MAF) < 0.01. Similarly, to the earlier processing of discovery cohort, quality control was carried on and only the filtered data was used for our analysis.

### Statistical Analyses

The demographic characteristics of the both ‘good responder’ and ‘poor responder’ groups were examined to confirm the homogeneity of the data used in our analysis. The data was found to have normal distribution, allowing student’s *t*-tests to be performed on the obtained data (age, PANSS score, etc.). Gender differences were analyzed using the Chi-square test. SPSS software (version 11.0, Chicago, IL, United States) was used for all the statistical analyses in this study. The association between genotype and OLA response was assessed using logistic regression model by PLINK vl.07 software (Purcell et al., 2007). *P*-values were corrected using Bonferroni method for multiple testing adjustments. Two-tailed *P*-values of 0.05 were considered to be statistically significant. Power analysis was performed by the software GPower 3.1.

## Results

### Patients Characteristics and Sequencing Profile

The demographic and clinical details of patient subjects included in this study are shown in Table 1. Among the 79 patients in the discovery cohort, 38 patients were defined to be good responders to OLA while 41 of them were poor responders. On the other hand, among 159 patients in independent cohort, 119 patients were good responders and 40 as poor responders to OLA. In the total cohort that comprised both sets, there were 157 good responders and 81 poor responders out of a total of 238 patient subjects. There was no statistically significant difference in the baseline characteristics between good responders and poor responders, except in the case of the PANSS total scores at the 8-week endpoint, meaning that the population was homogeneous (Table 1).

### Effects of Individual Polymorphisms on the OLA Response in the Discovery Cohort

Twelve out of 807 tested SNPs were found to be significantly associated with OLA response of 79 Han Chinese SCZ patients in discovery cohort. Table 2 lists the results of the SNP association analysis of pharmacogenetic impact on OLA treatment response (*P* < 0.05). Two newly found variants were located on the exon’s region (rs6280, *P* = 0.026, OR = 3.0, 95% CI = 1.14–7.87; rs2011404, *P* = 0.04, OR = 5.4, 95% CI = 1.08–26.93). The other 10 variants, which were not located in exons regions, were also found to be significantly associated with OLA response. However, there were no variants remained statistically significant after multiple-testing corrections (data not shown).

**Table 2:** 12 SNPs that were found to be significantly associated with responses to olanzapine treatment, by targeted sequencing.

Chr	SNP	Chr. Pos	MA	R_freq	NR_freq	OR (95% CI)	*P*	Gene	Annotation
2	rs4325692	211539363	A	0.07	0.00	NA	0.023	*CPS1*	Intronic
3	rs6280	113890815	C	0.31	0.18	3.0 (1.14–7.87)	0.026	*DRD3*	Non-synonymous
19	rs12610827	1538138	T	0.14	0.03	6.1 (1.23–30.30)	0.027	*PLK5* (neighboring gene)	Intergenic
14	rs7157205	43129681	C	0.20	0.07	3.9 (1.17–12.81)	0.027	*CTD-2307P3.1*	Intronic
7	rs1547470	151432869	T	0.14	0.04	4.9 (1.13–17.71)	0.032	*PRKAG2*	Intronic
3	rs324026	113891042	C	0.31	0.18	2.9 (1.08–7.54)	0.034	*DRD3*	Intronic
14	rs1543494	21829404	T	0.10	0.01	7.4 (0.86–63.22)	0.035	*SUPT16H*	Intronic
2	rs2011404	234627937	T	0.12	0.03	5.4 (1.08–26.93)	0.040	*UGT1A4*	Synonymous
19	rs2232778	8370120	T	0.27	0.39	0.3 (0.11–0.95)	0.040	*CD320*	3^′^UTR
8	rs4737005	39900682	T	0.08	0.20	0.3 (0.09–0.97)	0.045	*IDO2* (neighboring gene)	Intergenic
10	rs1831019	120691814	A	0.18	0.08	3.0 (1.01–9.02)	0.048	*LDHAP5* (neighboring gene)	Intergenic
10	rs9422807	126093991	T	0.03	0.11	0.23 (0.05–1.12)	0.049	*OAT*	Intronic


*Chr, chromosome; SNP, single-nucleotide polymorphism; Chr. Pos, chromosome position; MA, minor allele; R_freq, responder frequency; NR_freq, non-responder frequency; OR, odds ratio; CI, confidence interval*.

### Verification of the Genetic Variants Associated With the Response to OLA in the Independent and Total Cohort

We used independent cohort and total sample population composed of both discovery cohort and independent cohort to validate the 12 SNP signatures found from the analysis of discovery cohort. The relevant clinical information of the data is shown in Table 1. A total of 12 SNPs was genotyped from patients in these two sets. In particular, SNP rs324026 and rs12610827 were found to be significantly associated with OLA treatment response in the independent cohort (*P* = 0.023 and *P* = 0.023). In the combined cohort, 4 SNPs displayed significant difference in OLA response between good responders and poor responders. We obtained strong evidence to conclude that these 2 variants (rs324026 and rs6280) in the dopamine receptor D3 (*DRD3*) gene were significantly associated with OLA response in Han Chinese SCZ patients (*P* = 0.001 and 0.0047). In addition, SNP rs12610827 (near to *PLK5*) and rs1543494 (located in *SUPT16H*) were also shown to be significantly associated with the OLA treatment response (*P* = 0.001 and 0.038). Detailed information of these significant SNPs is shown in Table 3.

**Table 3:** Validation of SNPs associated with the olanzapine response.

										Functional
Chr	SNP	Chr. Pos	MA	Total cohort			Independent cohort		Gene	consequence
				OR (95% CI)	*P*	Adjust *P*^a^	OR (95% CI)	*P*		
3	rs324026	113891042	C	2.35 (1.41–3.93)	**0.001**	**0.014**	2.11 (1.1–4.0)	**0.023**	*DRD3*	Intronic
19	rs12610827	1538138	T	3.9 (1.81–8.45)	**0.00098**	**0.012**	2.49 (1.0–6.0)	**0.043**	*PLK5 (neighboring gene)*	–
3	rs6280	113890815	C	2.1 (1.25–3.46)	**0.0047**	0.057	1.85 (0.98–3.51)	0.058	*DRD3*	Missense
14	rs1543494	21829404	T	2.48 (1.05–5.86)	**0.038**	0.46	1.42 (0.55–3.67)	0.47	*SUPT16H*	Intronic


*Chr, chromosome; SNP, single-nucleotide polymorphism; Chr. Pos, chromosome position; MA, minor allele; OR, odds ratio; CI, confidence interval; ^a^P were adjusted by Bonferroni method. Bold font indicates statistically significant values*.

### Power Analysis

*Post hoc* power analysis revealed that the statistical power of the discovery cohort size (*n* = 79) in detecting a significant association (*P* < 0.05) was 0.76 with a medium effect size (Odds ratio = 2.0). The power of independent cohort size (*n* = 159) was 0.96 with the same effect size. These results indicated that the sample size in our study was sufficient to achieve a considerably low risk of a type II error.

## Discussion

To date, most pharmacogenomic studies on the OLA response focused on a few genes that are known to be relevant to OLA metabolism. Our study represents a more systematic survey of genetic biomarkers, including drug metabolic enzyme genes, receptor genes and other related genes. 143 genes of interest were sequenced using Next-generation sequencing technology for our association analysis. This study is one of the most comprehensive pharmacogenetic analyses of association between SNP variants and OLA response.

Our result suggested that SNP rs324026 in *DRD3* gene had significant association with OLA response using independent cohort. This difference still remained significant in the total cohort comprised 2 cohorts even after Bonferroni correction. However, the other variant rs6280 was only found to be significantly associated with an 8-week treatment of OLA response in the combined cohort population and did not have evidence to have significant associations with OLA response in independent cohort. This inconsistent result may be caused by the small sample set we tested. Notably, these 2 SNPs both exhibited strong linkage disequilibrium (*r*^2^ > 0.9) in the HaploReg database (Ward and Kellis, 2012). Therefore, both rs6280 and rs324026 may serve as biomarkers of OLA treatment response.

It is known that rs6280 mutation leads to a glycine for serine substitution and is associated with altered dopamine binding affinity. This glycine variant had been suggested to be able to increase the densities of the dopamine receptor D3 (DRD-3) in some areas in human brain (Jeanneteau et al., 2006). Adams et al. (2008) reported that DRD-3 gly/gly genotype and other polymorphisms in linkage disequilibrium with ser-9-gly variant were significantly associated with an increase in PANSS total score. Therefore, we concluded that our result was consistent with the earlier discoveries of the association between ser-9-gly variant and clozapine, which is the most similar receptor binding profile to OLA. Cerrato et al. (2017) surveyed 65 papers and found that rs6280 was successfully replicated as prognostic biomarkers of clozapine efficacy. In contrast, rs324026 variant had never been reported to affect the therapeutic efficacy of OLA. Rs324026 is located next to exon 5. Our analysis results suggested that we could only find evidence of significant association between SNP rs324026 and OLA efficacy after Bonferroni correction in the combined sample with both discovery and independent cohorts. Additionally, individuals with C alleles of rs324026 generally experience significantly better efficacy of OLA treatment.

In this study, SNP rs12610827 was validated in patients from independent cohort and its association with OLA response remained significant for multiple testing in the total cohort after Bonferroni correction. Rs12610827 variant is located near the *PLK5* gene. Polo-like kinases (Plks) family, consisted of 5 members (Plk1-Plk5), is traditionally regarded to play an important part only in cell cycle progression. However, mounting evidence showed that Plk2 and Plk5 are also closely involved in neuron biology (de Carcer et al., 2011a). It had been suggested that Plk2 modulates neurite formation in response to activities of brain-derived growth factor (BDGF) (Inglis et al., 2009). Additionally, Plk5 was highly expressed in the central nervous system and it serve as a Plk2-like role in the cerebellum according earlier report (de Carcer et al., 2011b). It had been suggested that Plk5 was regulated by CpG methylation of the promoter region on the transcriptional level (de Carcer et al., 2011a). The level of *PLK5* gene expression may be influenced by the methylation status of this variant. In this study, Han Chinese SCZ patients who carried allele T in SNP rs12610827 showed more good response in OLA treatment. However, no previous reports have found such association between *PLK5* variants and drug response. Therefore, we believe rs12610827 variant is worthy of further investigations in order to verify its influence on OLA response in the future.

This study has several limitations. First, our analysis did not consider some other genes that may have significant association with antipsychotic. Therefore, our results may have neglected some important biomarkers of OLA response due to this incomplete gene collection. Secondly, a number of identified SNP associations failed to stay statistically significant after Bonferroni corrections. This may be caused by over-correction because sample size was relatively small. Therefore, employing strict multiple corrections such as Bonferroni to the data may be too harsh for this specific study.

## Conclusion

In sum, we performed a comprehensive study on 238 Han Chinese SCZ patients in order to identify potential biomarkers of Han Chinese-specific OLA responses. The result showed that 143 genes were significantly associated with OLA. In addition, 2 variants (rs324026 and rs12610827) were found to have significant association with the OLA response. Future investigations with larger sample sizes and high-throughput methods such as high-density SNP arrays and whole exome sequencing are warranted to find more biomarkers to predict the efficacy of OLA in the Han Chinese population.

## Data Availability

The datasets used and/or analyzed during the current study are available from the corresponding author on reasonable request.

## Author Contributions

WZ and YX performed the experiments and wrote the manuscript. CH and ML aided in processing the data. QL, Y-hS, and YX aided in the collection of the materials. LC and LS helped in revising the manuscript. ZY, DC, and SQ designed and revised the manuscript.

## Conflict of Interest Statement

The authors declare that the research was conducted in the absence of any commercial or financial relationships that could be construed as a potential conflict of interest.

## Footnotes

1 https://www.pharmgkb.org/

2 www.agilent.com/genomics/suredesign

3 https://agenacx.com/
