# Supplementary material for: Genetic Association of Olanzapine Treatment Response in Han Chinese Schizophrenia Patients
Source: Front Pharmacol. 2019 Mar 4;10:177. doi: 10.3389/fphar.2019.00177 (PMC6409308; doi:10.3389/fphar.2019.00177)
Supplement: Supplementary file 1 [file Table_1.DOC]

**Supplementary Table 1.** 143 candidate genes involved in this study

| Classification | Gene names |
| --- | --- |
| Drug transport enzymes | *ABCB1, ABCC2,* *ATP7B, SLC17A3, SLC25A13, SLC25A15, SLC37A4,* *CPT1A* |
| Drug-metabolizing enzyme | *ACACA,* *ALDH18A1,* *ALDOA,* *AGL,* *ARG1,* *ARSB,* *ASS1,* *BCKDHA、BCKDHB,* *BTD,* *CDKL5, COMT, CPS1, CPT1A,* *CYP1A2, CYP2D6, CYP3A4,* *DAO,* *DBT,* *DDC,* *DLD,* *ENO3, FAH,* *GALNS, GAA, GBA, GBE1, GLA, GLB1, GLUD1, G6PC, GNB3, GNS, GSTZ1, GUSB, GYG1, GYS(1,2), HGSNAT, HLCS, HNF1A, HPD, HYAL1, HTT, MECP2, IDS, IDUA, LDHA, LMBRD1, MCEE, MLYCD, MMAA, MMAB, MMA(C,D)HC,* *MUT, NAGLU,* *NAGS, OAT, OTC, PC, PFKM, PGAM2, PGM1, PHKA(1,2),* *PHKB,* *PHKG2, PRKAG2,* *PRNP, PSAP,* *PYGL,* *SUCLA2, SUCLG1,* *SUMF1,* *TAT, UGT1A(1,3,4,5,6,7,8,9,10), UGT2A(1,2,3), UGT3A(1,2), UGT2B(4,7,10,11,15,17,28)* |
| Drug-targeted receptors | *ADRA1A, ADRA1B, ADRA1D, ADRA2A, ADRA2B, ADRA2C, ADRB1, ADRB2, ADRB3,* *CD320, CHRM(1,2,3,4,5), DRD(1,2,3,4,5), GABRA(1,5,6), GABRB(2,3), GABRG(2,3), GRIK2, GRIN1, GRIN2(A,B,C,D), HTR(1,2)A, HTR2(B,C), HTR3A, HTR6,* |

**Supplementary Table 2.** Primer sequence of MassARRAY validation

| SNP ID | Forward Primer (5'-3') | Reverse Primer (5'-3') | Extended Primer (5'-3') |
| --- | --- | --- | --- |
| rs12610827 | ACGTTGGATGCGCACAAAAGGGCATCATTG | ACGTTGGATGAAGAATGGCGTGAACCCAG | GGCATCATTGACATCTGGAG |
| rs1543494 | ACGTTGGATGTGATGCTCGGTATGTACTGC | ACGTTGGATGATCAGTACAATTTCCCTGGC | attgCCCTGGCCTGAAAAAG |
| rs1547470 | ACGTTGGATGACGTATTCCTCTAACCACCG | ACGTTGGATGTGTGTGCACACCCTCCTAAG | ACCACCGCCTGATGTTATA |
| rs1831019 | ACGTTGGATGCAATTATGCACAAGACATGCG | ACGTTGGATGCTGAACATGCCTAGTCCAAC | GCCTAGTCCAACATTTTTT |
| rs2011404 | ACGTTGGATGTGTGGTTTTAACAGACCCCG | ACGTTGGATGCACATGGAATGTACCTCCAA | CCCCGTTAACCTCTG |
| rs2232778 | ACGTTGGATGTCTCCAAGAGCACAGTAGTC | ACGTTGGATGAGTGTGGTTCCTATATCCCG | GCCTGTCTCCTGCGG |
| rs324026 | ACGTTGGATGAGGCACTGTTGTGAGAGTTG | ACGTTGGATGAGAATGGGAGCTTCAAAGGG | GCAATGAACAAAACAGATAAAAT |
| rs4325692 | ACGTTGGATGGTAGAACCGAAGTGGATCTG | ACGTTGGATGCCCTGCTTTTCACCTTGTTC | TTATCTCCTTTTATTCTCTATTTAATC |
| rs4737005 | ACGTTGGATGCAAGACCCTATCTCAGAAAC | ACGTTGGATGCAAGAGTGAGGCTCTCAAAC | CAACAAAACCCCACAAA |
| rs6280 | ACGTTGGATGTGGCACCTGTGGAGTTCTCT | ACGTTGGATGTCTGGGCTATGGCATCTCTG | AGGTGTAGTTCAGGTGGC |
| rs7157205 | ACGTTGGATGCGACAGAGAGACTCCATCAA | ACGTTGGATGTTCTTTTCTACACAGCTCAC | CACAGCTCACTTTTGTTC |
| rs9422807 | ACGTTGGATGTCTATTCCCAATGAGCTGAG | ACGTTGGATGTTATGCCGGGATTCGACATC | TGAGCTGAGCACTATGA |
